# Transfer of gene-corrected T cells corrects humoral and cytotoxic defects in patients with X-linked lymphoproliferative disease

**DOI:** 10.1016/j.jaci.2018.02.053

**Published:** 2018-07

**Authors:** Neelam Panchal, Ben Houghton, Begona Diez, Sujal Ghosh, Ida Ricciardelli, Adrian J. Thrasher, H. Bobby Gaspar, Claire Booth

**Affiliations:** a Molecular and Cellular Immunology Section, UCL GOS Institute of Child Health, London, United Kingdom; b Department of Pediatric Oncology, Hematology and Clinical Immunology, Medical Faculty, Center of Child and Adolescent Health, Heinrich-Heine-University, Dusseldorf, Germany; c Department of Paediatric Immunology, Great Ormond Street Hospital NHS Trust, London, United Kingdom

**Keywords:** X-linked lymphoproliferative disease, T-cell gene therapy, follicular helper T cells, T-cell cytotoxicity, BV, Brilliant Violet, CTL, Cytotoxic T lymphocyte, HLH, Hemophagocytic lymphohistiocytosis, HSCT, Hematopoietic stem cell transplantation, HVS, *Herpesvirus saimiri*, LCL, Lymphoblastoid cell line, MOI, Multiplicity of infection, NK, Natural killer, NP-CGG, 4-hydroxy-3-nitrophenylacetly conjugated chicken gammaglobulin, PD-1, Programmed cell death protein 1, PE, Phycoerythrin, SAP, SLAM-associated protein, T_FH_, T follicular helper, XLP, X-linked lymphoproliferative disease

## Abstract

**Background:**

X-linked lymphoproliferative disease 1 arises from mutations in the *SH2D1A* gene encoding SLAM-associated protein (SAP), an adaptor protein expressed in T, natural killer (NK), and NKT cells. Defects lead to abnormalities of T-cell and NK cell cytotoxicity and T cell–dependent humoral function. Clinical manifestations include hemophagocytic lymphohistiocytosis, lymphoma, and dysgammaglobulinemia. Curative treatment is limited to hematopoietic stem cell transplantation, with outcomes reliant on a good donor match.

**Objectives:**

Because most symptoms arise from defective T-cell function, we investigated whether transfer of SAP gene–corrected T cells could reconstitute known effector cell defects.

**Methods:**

CD3^+^ lymphocytes from Sap-deficient mice were transduced with a gammaretroviral vector encoding human *SAP* cDNA before transfer into sublethally irradiated Sap-deficient recipients. After immunization with the T-dependent antigen 4-hydroxy-3-nitrophenylacetly chicken gammaglobulin (NP-CGG), recovery of humoral function was evaluated through germinal center formation and antigen-specific responses. To efficiently transduce CD3^+^ cells from patients, we generated an equivalent lentiviral SAP vector. Functional recovery was demonstrated by using *in vitro* cytotoxicity and T follicular helper cell function assays alongside tumor clearance in an *in vivo* lymphoblastoid cell line lymphoma xenograft model.

**Results:**

In Sap-deficient mice 20% to 40% engraftment of gene-modified T cells led to significant recovery of germinal center formation and NP-specific antibody responses. Gene-corrected T cells from patients demonstrated improved cytotoxicity and T follicular helper cell function *in vitro*. Adoptive transfer of gene-corrected cytotoxic T lymphocytes from patients reduced tumor burden to a level comparable with that seen in healthy donor cytotoxic T lymphocytes in an *in vivo* lymphoma model.

**Conclusions:**

These data demonstrate that autologous T-cell gene therapy corrects SAP-dependent defects and might offer an alternative therapeutic option for patients with X-linked lymphoproliferative disease 1.

X-linked lymphoproliferative disease 1 (XLP1) is a severe primary immunodeficiency arising from mutations in the *SH2D1A* gene, which encodes an intracellular adaptor protein called SLAM-associated protein (SAP). The absence of SAP leads to multiple immunologic defects, including impaired T-cell and natural killer (NK) cell cytotoxicity,^1^, ^2^, ^3^, ^4^ lack of NK T-cell development,^5^, ^6^ and defective CD4^+^ T follicular helper (T_FH_) cell help,^7^, ^8^, ^9^ which leads to abnormal humoral function. The clinical disease phenotype is characterized by severe immune dysregulatory phenomena, including abnormalities in immunoglobulin production and T-dependent humoral immune responses, T-cell effector defects leading to hemophagocytic lymphohistiocytosis (HLH), and development of lymphoma.

Specific disease manifestations can be treated supportively with replacement immunoglobulin for dysgammaglobulinemia, HLH chemotherapeutic protocols, monoclonal serotherapy for EBV-driven disease, and appropriate chemotherapy regimens for malignancy, but curative treatment for patients with XLP1 is limited to allogeneic hematopoietic stem cell transplantation (HSCT). Results are highly dependent on a good donor match and the absence of active disease at transplantation, with survival decreasing to 50% if patients enter transplantation with HLH.^10^ For more than 2 decades, autologous hematopoietic stem cell gene therapy has been shown to be a successful treatment option for specific immune deficiencies,^11^ and this experience supports the development of therapeutic gene therapy strategies for other monogenic immune deficiencies.

In a Sap-deficient mouse model we demonstrated correction of cellular and humoral defects through lentivirus-mediated gene transfer into hematopoietic progenitor cells, thereby providing proof of concept for gene therapy in patients with XLP1.^12^ One concern about this approach was that the nonphysiologic expression of SAP in progenitor cell populations after stem cell gene transfer might be associated with certain risks because of the role of SAP as an important signaling molecule and its tightly regulated expression profile. Although no adverse effects were seen when SAP was expressed in HSCs or other hematopoietic compartments in which expression is usually limited, we wanted to evaluate whether transfer of gene-corrected T cells can offer a potentially safer treatment option. We evaluated a number of regulatory elements in the context of a hematopoietic stem cell gene therapy approach to provide lineage-specific SAP expression but were unable to identify a promoter capable of affording specificity and sufficient protein expression to restore immune function (unpublished data).

Autologous T-cell gene therapy would diminish concerns over ectopic SAP expression and has an established safety profile, with hundreds of patients treated to date for hematologic malignancies in cancer immunotherapy trials and no reported transformational events.^13^, ^14^, ^15^, ^16^, ^17^ Furthermore, important manifestations of XLP1, such as HLH, lymphoma development, and dysgammaglobulinemia, arise from defective T-cell function and would be potentially corrected through this approach. Therefore we sought to investigate whether infusion of gene-modified T cells could correct both humoral and cytotoxic immune defects in a Sap-deficient murine model and an *in vivo* tumor model by using corrected cells from patients. Here, for the first time, we show that viral vector–mediated gene correction of the T-cell compartment can recover these immune defects both *in vitro* and *in vivo*. This work provides evidence that an autologous gene-corrected T-cell approach might offer therapeutic benefit to patients with XLP1.

## Methods

### Mice

Sap-deficient mice (*Sap*^y/−^, *Sap*^−/−^) have been previously described.^18^, ^19^, ^20^ NSG (NOD/SCID/IL-2Rγ^null^) mice were obtained from the Jackson Laboratory (Bar Harbor, Me). Animals were raised in specific pathogen-free conditions, and all studies were licensed under the Animals (Scientific Procedures) Act 1986 (Home Office, London, United Kingdom).

### Vector constructs

For murine experiments, a gammaretroviral vector on the SF91 backbone containing codon-optimized human *SAP* cDNA with an internal ribosomal entry site element and enhanced green fluorescent protein (eGFP) was used. Human primary cell experiments were carried out by using a third-generation lentiviral vector on a pCCL backbone containing codon-optimized human *SAP* cDNA driven by the elongation factor 1α short (EFS) promoter, internal ribosomal entry site, and eGFP or eGFP alone (EFS-SAP-eGFP; EFS-eGFP).

### Murine CD3^+^ T-cell selection and transduction

CD3^+^ T cells were isolated by means of negative magnetic selection (pan-T cells; MicroBeads; Miltenyi Biotec, Bergisch Gladbach, Germany) from harvested splenocytes and cultured in RPMI 1640, 10% FCS, 1% penicillin/streptomycin, 1 mmol/L β-mercaptoethanol, and 1 mmol/L sodium pyruvate (all from Life Technologies, Grand Island, NY) and stimulated with 20 U/mL murine IL-2 (PeproTech, Rocky Hill, NJ) and 100 μg/mL anti-CD3e and 100 μg/mL anti-CD28 T-cell activation/expansion (Miltenyi Biotec).

Transduction was performed 24 hours later with a retroviral supernatant using spinoculation (90 minutes at 1000*g*) in recombinant human fibronectin fragment (RetroNectin; Takara Bio Europe S.A.S, Saint-Germain-en-Laye, France)–precoated plates. Seventy-two hours after transduction, cells were harvested and analyzed by flow cytometry (LSRII; BD, San Jose, Calif) for transduction efficiency and the CD4^+^/CD8^+^ phenotype by using rat anti-mouse CD8a (allophycocyanin) and rat anti-mouse CD4 (Brilliant Violet [BV] 510; BD Biosciences). CD3^+^ cells were injected into sublethally (6 Gy) irradiated Sap-deficient recipient mice. Eight weeks after reconstitution, mice were challenged through intraperitoneal injections of T cell–dependent antigen: chicken gammaglobulin conjugated to the hapten 4-hydroxy-3-nitrophenylacetly (NP-CGG; 150 μg/mL).

### Patient samples

Consent was obtained to use samples from 4 unrelated patients. All patients had proven mutations in *SH2D1A* (P1, c.57_59 duplication; P2, hemizygous deletion of exon 2; P3, large deletion spanning from exon 2; and P4, large deletion spanning exons 2-4). Samples from P1, P2, and P4 were used for T_FH_ cell assays, and samples from P1, P3, and P4 were used in cytotoxicity experiments.

### *In vitro* T_FH_ cell assay

PBMCs were isolated by using Ficoll centrifugation (GE Healthcare, Amersham, United Kingdom). Naive CD4^+^ T cells were isolated from nontransformed healthy donor PBMCs and *Herpesvirus saimiri* (HVS)–transformed samples from patients with XLP1 using Miltenyi MACS-negative selection, according to the manufacturer's protocol. Selected cells were activated by using either anti-CD3/CD28 Dynabeads at a 1:1 ratio with X-VIVO media (Sigma, St Louis, Mo) supplemented with 5% human serum and human recombinant IL-2 at a concentration of 20 U/mL or with human recombinant IL-6 (100 U/mL), IL-7 (20 U/mL), or IL-21 (200 U/mL). Cells were incubated for 72 hours and transduced at a multiplicity of infection (MOI) of 50. Cells were cultured subsequently, maintaining *in vitro* conditions at a density of 5.0 × 10^4^ cells/well of a 96-well round-bottom plate in the presence of 15 ng/mL staphylococcal enterotoxin B either alone or with allogeneic memory B cells isolated from tonsillar mononuclear cells at a ratio of 1:1. Cells were cocultured for 10 days before immunophenotyping with LSRII and BD-conjugated antibodies: mouse anti-human CD4 (BV650), mouse anti-human programmed cell death protein 1 (PD-1; CD279; BV510), and mouse anti-human CXCR5 (CD189; BV41). ELISAs to analyze IL-21 and IgM/IgG/IgG_1-3_ concentrations were performed on supernatants by using Platinum ELISA kits (eBioscience, Carlsbad, Calif).

### Generation of lymphoblastoid cell lines and cytotoxic T lymphocytes

PBMCs were isolated as above from EBV-seropositive healthy donors for generation of autologous and allogeneic lymphoblastoid cell lines (LCLs) by using the EBV B95.8 supernatant (kindly provided by Ida Ricciardelli) in the presence of 50 μg of cyclosporine. PBMCs were stimulated with 40 Gy of irradiated LCLs *in vitro* over a period of 4 weeks with weekly stimulation.

### *In vitro* cytotoxicity and cytotoxic T-lymphocyte phenotyping

Generated cytotoxic T lymphocytes (CTLs) were transduced by using EFS-SAP-eGFP or EFS-eGFP vectors at an MOI of 50 and phenotyped by using phycoerythrin (PE)–labeled mouse anti-CD4, allophycocyanin-labeled mouse anti-CD8, BV650-labeled mouse anti-human CD45RA, and BV510-labeled mouse anti-human CD62L (BD Biosciences) to establish memory phenotype and transduction efficiency before the NSG tumor model. CTL function was determined by using an *in vitro* ^51^Cr (Na_2_^51^CrO_4_; PerkinElmer, Waltham, Mass) release assay with allogeneic LCL targets and nonspecific P185 murine mastocytoma cell targets with anti-CD3 conjugation (BD Biosciences) to determine EBV-directed and redirected killing. An effector/target ratio of 30:1 was used, and serial dilutions were performed to determine the cytotoxicity range and incubated for 4 hours at 37°C. ^51^Cr release in the supernatant was measured with a beta counter (Trilux, 1450 MicroBeta; PerkinElmer).

### NSG tumor model

Allogeneic LCLs were transduced with a lentiviral vector expressing firefly luciferase and blue fluorescent protein reporter gene to select on highly expressing cells. A cell dose of 5.0 × 10^6^ was prepared in Matrigel Matrix (Corning, Corning, NY) with equal amounts of PBS and injected into NSG mice subcutaneously at the nape of the neck. Forty-eight hours after LCL injections, xenografted tumor burden was established by using the IVIS *in vivo* imaging system (Xenogen; Caliper Life Sciences, Hopkinton, Mass) in the presence of 15 mg/kg intraperitoneally injected D-Luciferin. CTLs from healthy donors and patients with XLP1 were injected intravenously through the tail vein at a 1:1 CTL/LCL ratio (5 × 10^6^ total cells per mouse), and tumor regression was monitored by using IVIS every 48 hours for 10 days after tumor establishment.

### Statistical analysis

Statistical analysis was performed with GraphPad Prism 6.0 software (GraphPad Software, La Jolla, Calif). Statistical significance for murine experiments was determined by using 2-way ANOVA. Statistical significance for cytotoxicity and T_FH_ cell *in vitro* assays was calculated by using appropriate nonparametric testing, including Sidak, Dunnett, and Kruskall-Wallis multiple-comparison tests and Mann-Whitney 2-tailed Student *t* tests assuming non-Gaussian distribution.

## Results

### Transfer of SAP-replete T cells restores humoral immunity

**Fig 1:**
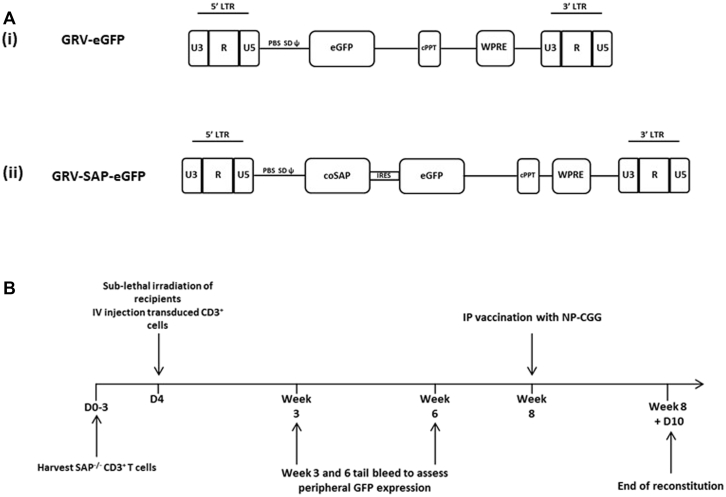
Experimental design of the T-cell adoptive transfer model. **A,** Schematic representation of long terminal repeat *(LTR)*–driven gammaretroviral vector used for *ex vivo* transduction of murine donor splenic CD3^+^ selected T cells. *i*, Mock vector containing eGFP reporter gene only. *ii*, Vector containing codon-optimized SAP cDNA and eGFP. **B,** Timeline of adoptive T-cell transfer experiments after sublethal (6 Gy) irradiation at day 0 with intravenous infusion of transduced murine CD3^+^ T cells. Animals underwent tail vein bleeds at 3 and 6 weeks to assess peripheral T-cell donor engraftment before immunologic challenge at week 8 with the T cell–dependent antigen NP-CGG.

**Fig E1:**
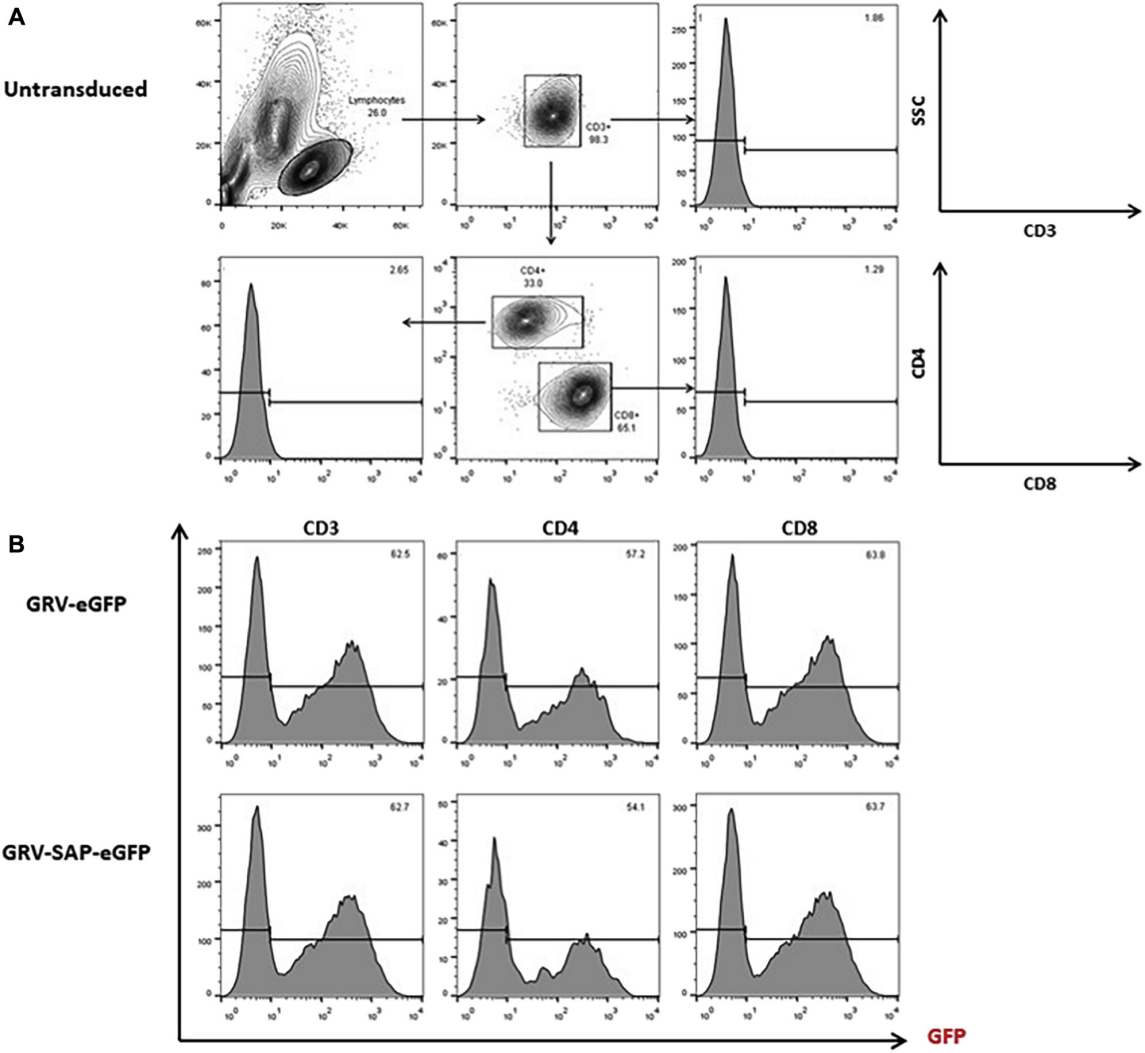
Transduction efficiency and phenotype of murine splenic CD3^+^ donor T cells. **A,** Flow cytometric staining of murine T cells showing the gating strategy for CD4^+^ and CD8^+^ lymphocyte populations. **B,** Transduction efficiencies are determined by using GFP expression 72 hours after transduction with GRV-SAP-eGFP or GRV-eGFP alone. An average transduction of 50% to 60% is observed across T-lymphocyte compartments.

To demonstrate that SAP expression in the T-cell compartment can correct humoral immunity in a Sap-deficient murine model, we adoptively transferred gene-corrected Sap-deficient CD3^+^ T cells into Sap-deficient recipients after sublethal irradiation (6 Gy). A retroviral construct was used to transduce T cells in this context because murine T cells are not transduced efficiently with lentiviral vectors due to restriction factors present in this population.^21^ We generated gammaretroviral vectors containing a codon-optimized human *SAP* cDNA and eGFP or eGFP alone (Fig 1, *A*) and efficiently transduced Sap-deficient CD3^+^ lymphocytes with a standard activation protocol by using anti-CD3/CD28 beads and IL-2 (approximately 60% transduction efficiency for both SAP and GFP control vectors with an average vector copy number of 2.4). As expected, there was a slight predominance of CD8^+^ cells in transduced populations, but high levels of transduction were achieved in both the CD4^+^ and CD8^+^ compartments (see Fig E1 in this article's Online Repository at www.jacionline.org). Transduced cells from female Sap-deficient donors were adoptively transferred into male Sap-deficient recipients after sublethal irradiation to determine T-cell engraftment levels. At 8 weeks after infusion of gene-modified T cells, animals were challenged with NP-CGG and analyzed 10 days later to assess response to immunization (Fig 1, *B*).

**Fig 2:**
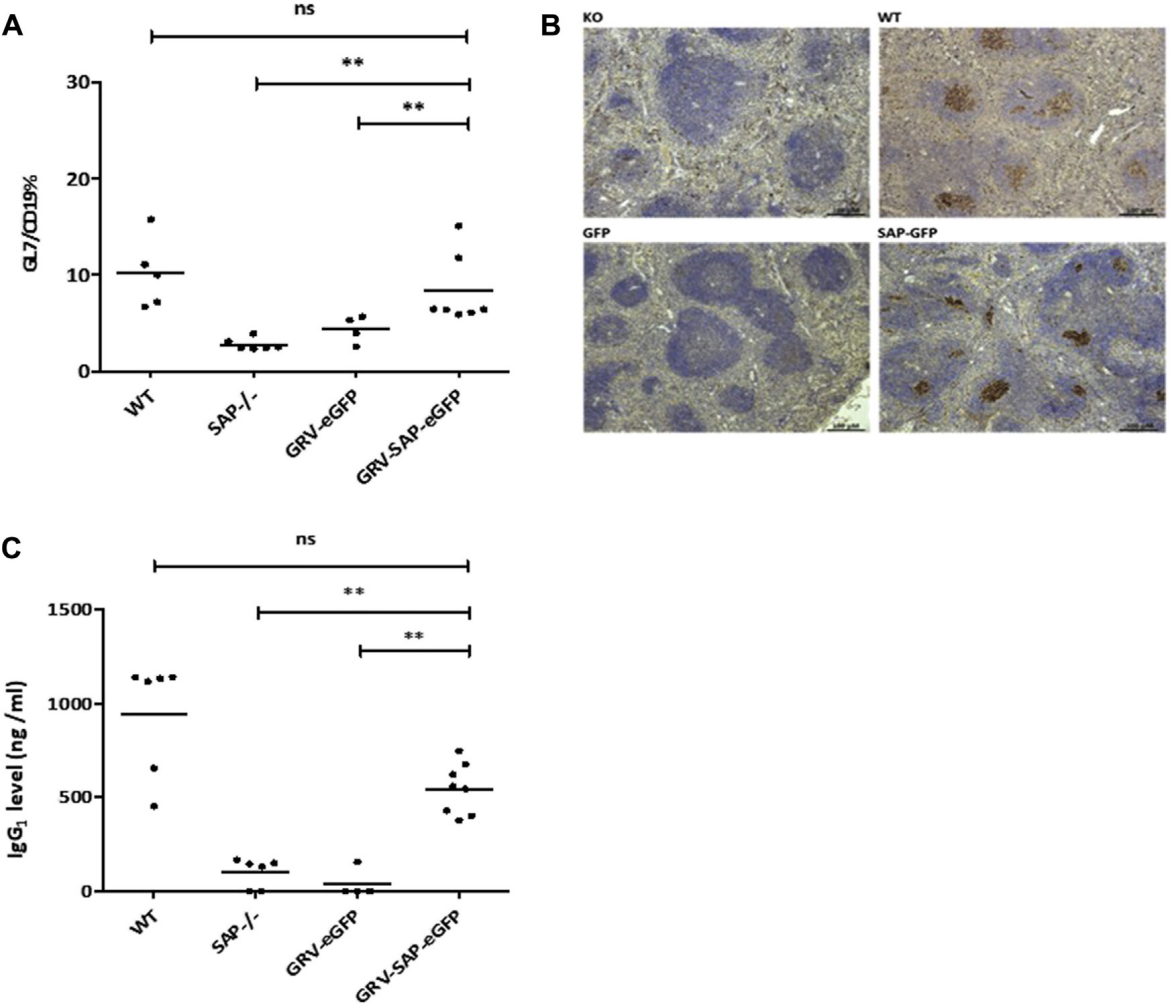
Analysis of humoral function after immunologic challenge. **A,** Analysis of germinal center B cells using flow cytometry in splenic lymphocytes stained with anti-CD19 and anti-GL7 antibodies. Results from individual mice are represented by *dots*, with mean values represented by a *horizontal bar*. **B,** Germinal center staining in splenic follicles using peanut agglutinin marking for germinal center B cells 10 days after immunization with NP-CGG at a magnification of ×40. Slides are representative of results seen in wild-type *(WT)* and Sap-deficient control animals and animals receiving gene-modified T cells (transduced with GRV-eGFP or GRV-SAP-eGFP vectors) demonstrating recovery of germinal centers in Sap-deficient mice receiving SAP-containing vector. **C,** NP-specific antibody production analysis with an ELISA performed on serum samples of all cohorts after NP-CGG vaccination, demonstrating functional restoration of germinal center activity in SAP-reconstituted animals comparable with that of wild-type littermates. **P < .05. *ns*, Not significant.

**Fig E2:**
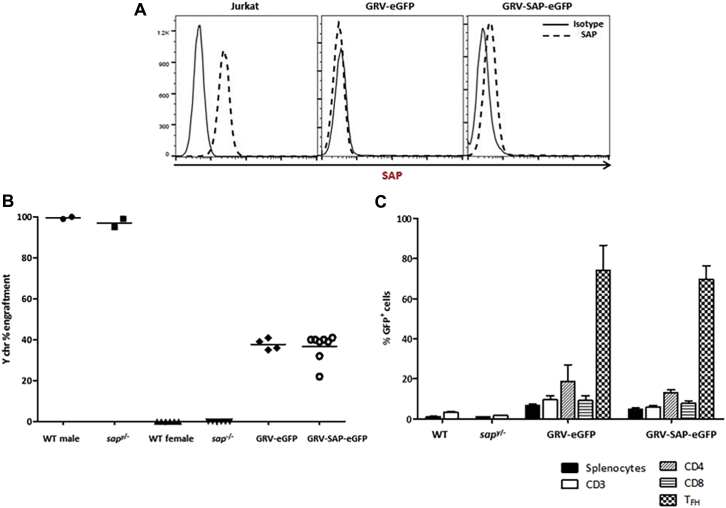
Analysis of adoptively transferred T cells. **A,** SAP protein expression was analyzed in gene-corrected murine splenic CD3^+^ T cells after reconstitution. **B,** Donor T-cell engraftment was assessed in whole splenocytes by using XY PCR techniques 10 weeks after reconstitution, showing average engraftment levels of 40%. **C,** Analysis of GFP levels in whole splenocytes and splenocyte-derived T-cell lineages in immunized animals.

We demonstrated significantly higher levels of GL7^+^/CD19^+^ cells (a germinal center marker) in the spleens of animals receiving the GRV-SAP-eGFP vector compared with those receiving the control eGFP vector (*P* < .01) with levels comparable with those in wild-type animals (Fig 2, *A*). Germinal center formation was confirmed by means of peanut agglutinin staining of splenic sections in Sap-deficient animals receiving gene-corrected T cells (Fig 1, *B*). We also found significantly improved NP-specific IgG_1_ levels (*P* < .01) in the GRV-SAP-eGFP vector–treated group in comparison with those seen in animals in the Sap-deficient or eGFP control group, demonstrating functional humoral reconstitution after correction of the T-cell compartment (Fig 1, *C*, and see Fig E2 in this article's Online Repository at www.jacionline.org). Higher eGFP levels were seen in the T_FH_ cell population of immunized animals receiving both vectors (see Fig E2, *C*), which is consistent with expansion of this population in response to antigen challenge, confirming that SAP is required only for T_FH_ cell function and not development. Together, these data suggest that infusion of gene-corrected Sap-deficient T cells can lead to restoration of functional humoral responses to T-dependent antigens.

### SAP gene transfer restores T_FH_ cell function

**Fig 3:**
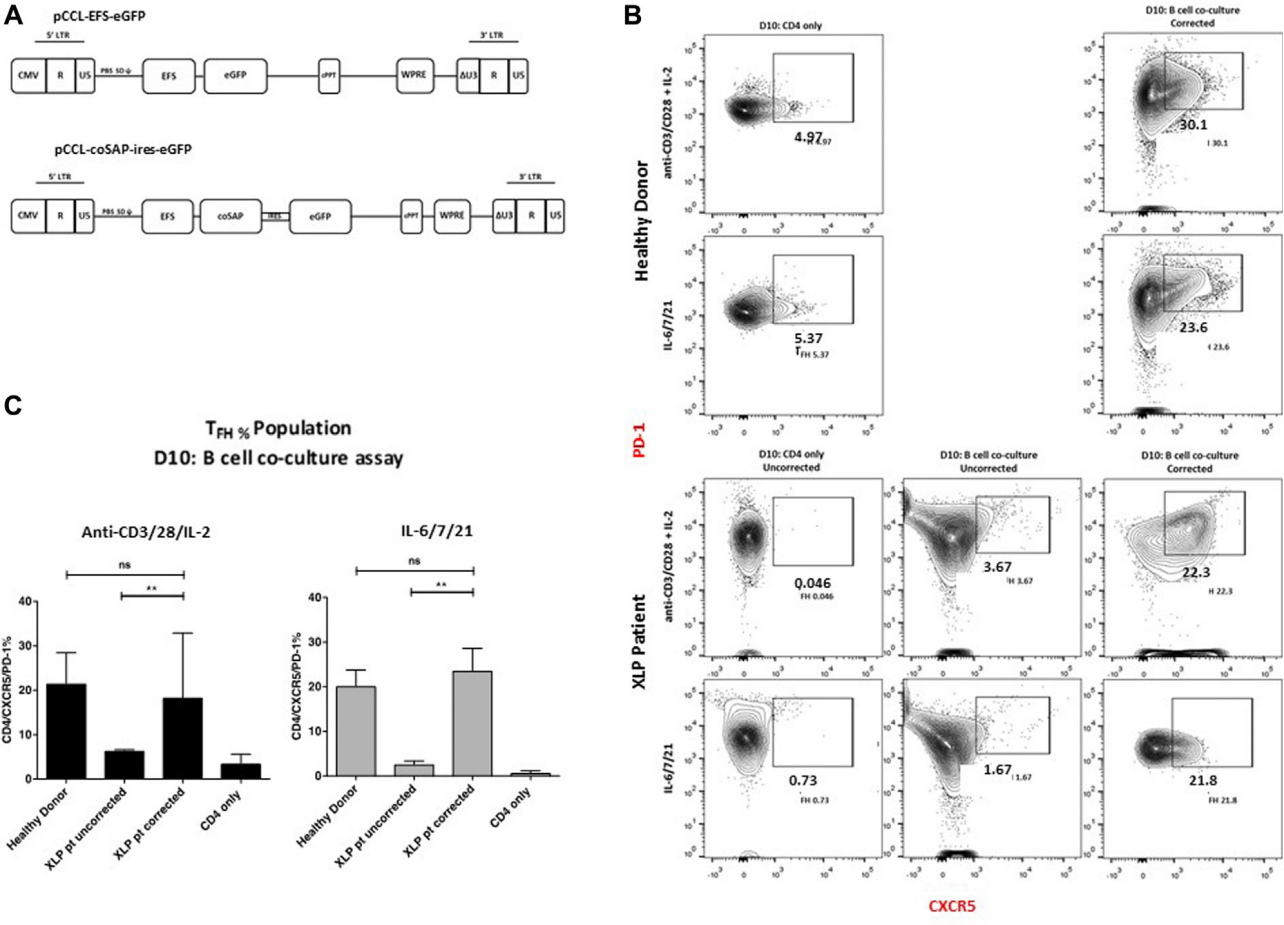
Correction of PBMC-derived T_FH_ cells from patients with XLP1. **A,** Schematic representation of lentiviral vectors containing codon-optimized human SAP cDNA driven by the EFS promoter and eGFP or EFS-eGFP only. An MOI of 20 was used to transduce PBMC-derived selected naive CD4^+^ cells before *in vitro* B-cell coculture assay. Transduction efficiency ranged between 30% to 45% (data not shown), with a vector copy number of 2 to 3 copies per cell. **B,** Representative flow cytometric contour plots of the differentiated CD4^+^ cell phenotype 10 days after coculture with allogeneic tonsillar memory B cells or CD4 cell culture alone. *Top 2 panels (middle to right)*, Healthy donor cells prestimulated with standard activation conditions (1.0 × 10^6^ cells/mL) with a 1:1 anti-CD3/CD28 bead ratio plus human IL-2 (10 ng/mL) or the T_FH_ cell–polarizing cytokines IL-6 (100 ng/mL), IL-7 (10 ng/mL), or IL-21 (20 ng/mL). *Bottom 2 panels (left to right)*, PBMC-derived and HVS-transformed differentiated CD4^+^ T cells from patients with XLP1 by using the conditions as above. Cells were transduced 3 days after stimulation and cultured with or without allogeneic B cells. **C,** Recovery of the T_FH_ cell population, as determined based on CXCR5 and PD-1 expression in corrected cells from patients with XLP1 after B-cell coculture assay in cells cultured in both anti-CD3/CD28/IL-2 and IL-6/IL-7/IL-21 culture conditions (n = 3). **P < .05. *ns*, Not significant.

**Fig E3:**
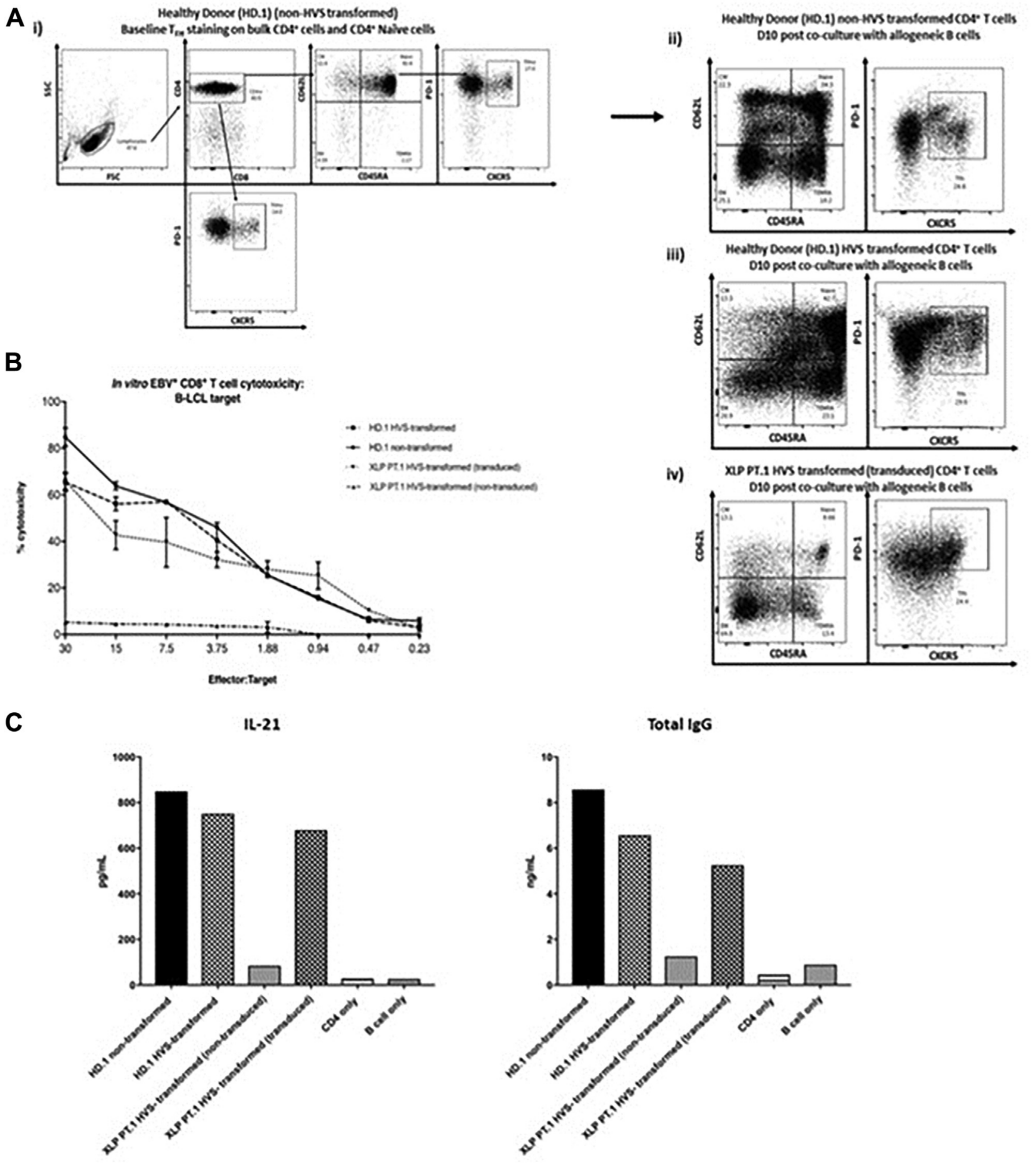
Comparison of function between HVS-transformed cells and untransformed cells. One experiment was performed comparing phenotype **(A)**, cytotoxicity **(B)**, and T_FH_ cell function **(C)** in HVS-transformed cells from healthy donors, HVS-transformed cells from patients, and nontransformed healthy donor cells, which demonstrated comparability among the all samples. No significant differences were observed in both cytotoxicity and T_FH_ cells assays.

To explore the clinical relevance of the data generated in the Sap-deficient murine model, we investigated whether restoration of SAP expression in T_FH_ cells from patients with XLP1 could ameliorate defective T_FH_ cell activity using an established *in vitro* T_FH_ cell functional assay.^22^ In patients with XLP1, the T_FH_ cell population secreted less IL-21, which is indicative of T_FH_ cell dysfunction, and B cells did not secrete immunoglobulins. For transduction of human primary T cells, we used a lentiviral vector with SAP expression driven by the EFS promoter (Fig 3, *A*). This vector had been used previously in our proof-of-concept hematopoietic stem cell gene therapy studies.^12^ Given the scarcity of primary patient samples, we generated T-cell lines from patients through transformation with HVS. We were able to demonstrate that the function of HVS-transformed lymphocytes was comparable with that of nontransformed T cells in terms of cytotoxicity and memory phenotype (see Fig E3 in this article's Online Repository at www.jacionline.org).

**Fig 4:**
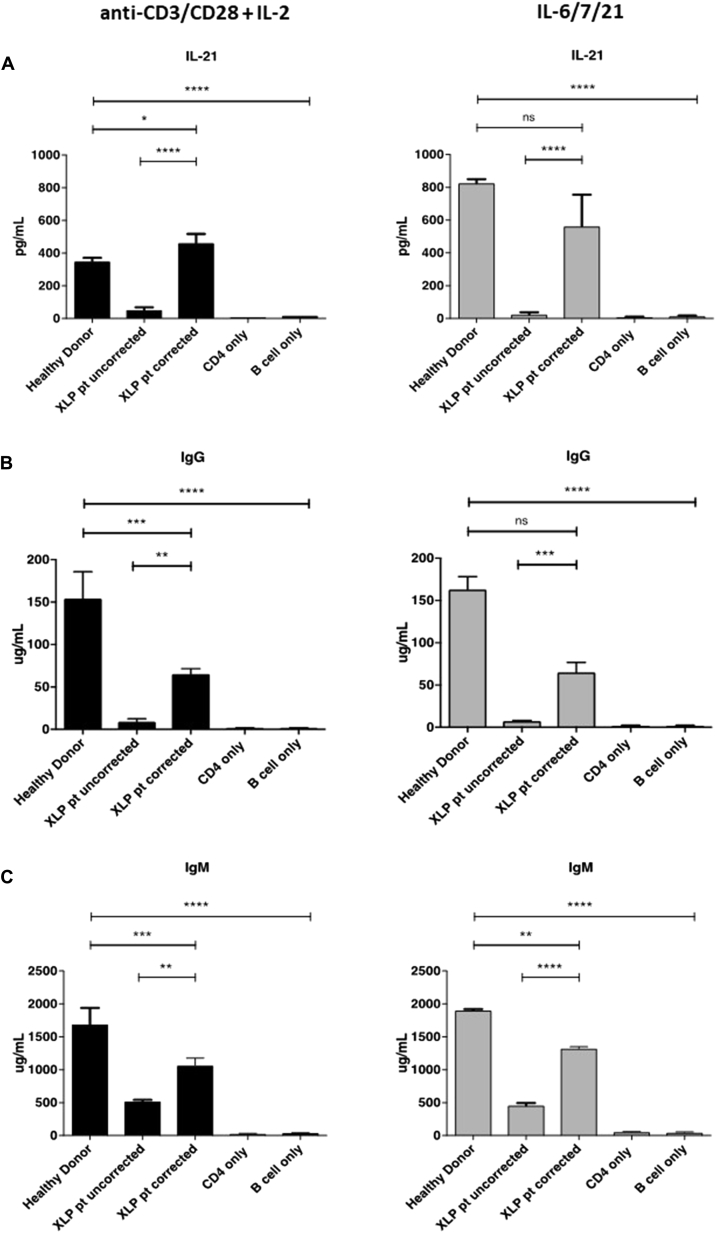
Functional correction of T_FH_ cells from patients with XLP1 *in vitro*. Quantification of IL-21 concentrations **(A)** and IgG **(B)** and IgM **(C)** levels in supernatants 10 days after coculture of naive CD4^+^ T cells and allogeneic B cells by means of ELISA. **P < .05, ***P < .001, and ****P < .0001. *ns*, Not significant.

**Fig E4:**
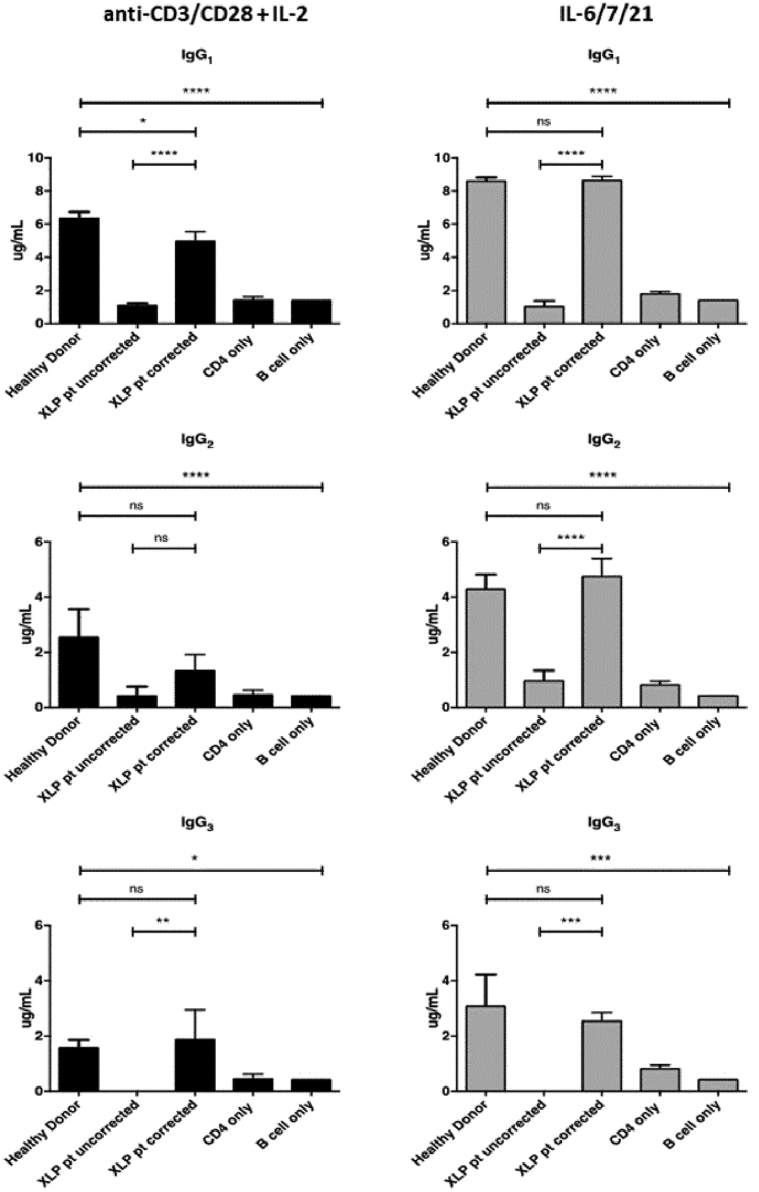
Functional correction of T_FH_ cells from patients with XLP1 *in vitro*: IgG subclass production. Quantification of IgG subclass levels in supernatants 10 days after coculture of naive CD4^+^ T cells and allogeneic B cells by means of ELISA. **P < .05, ***P < .001, and ****P < .0001. *ns*, Not significant.

Naive CD4^+^CD45RA^+^ cells from both HVS-transformed cells from patients and PBMCs from healthy donors (untransformed) were selected and then cultured in 2 different conditions to determine whether maintenance and transduction of the T_FH_ cell population could be optimized through cytokine stimulation. A standard activation protocol using anti-CD3/CD28 Dynabeads and human recombinant IL-2 at 100 U/mL was compared with a predicted pro-T_FH_ cytokine cocktail of IL-6 and IL-21^23^, ^24^, ^25^, ^26^, ^27^, ^28^ with a media supplement of IL-7 to allow proliferation of cells before lentiviral transduction with either EFS-eGFP or EFS-SAP-eGFP vectors. Transduced cells (transduced at 35% to 40% efficiency determined by using eGFP expression) were either cultured alone or cocultured with allogeneic memory B cells derived from healthy donor tonsillar samples at a ratio of 1:1 for a 10-day incubation period. Cells were then analyzed to determine the overall T_FH_ cell percentage (CD4^+^CXCR5^+^PD-1^+^; Fig 3), and the supernatant was tested to quantify the concentration of secreted IL-21 and immunoglobulin levels (Fig 4). The T_FH_ cell population was significantly reduced in patients with XLP1 compared with that in healthy control subjects (Fig 3, *B* and *C*), and this was associated with lower levels of IL-21 secretion and immunoglobulin production (Fig 4, *A* and *B*). In contrast, we found that the T_FH_ cell population, as characterized by CD4^+^CXCR5^+^PD-1^+^ expression, was restored to normal levels when cells from patients with XLP were gene corrected with SAP-expressing vector (Fig 3, *B* and *C*). There was no difference in the percentage of the T_FH_ cell population between cells transduced with anti-CD3/CD28 Dynabeads or with a pro-T_FH_ cytokine cocktail of IL-6 and IL-21. We also demonstrated functional recovery of the T_FH_ cell population in gene-corrected cells from patients with XLP1 with significantly increased IL-21 secretion, IgM levels, and IgG levels (including IgG_1_, IgG_2_, and IgG_3_ subclasses, *P* < .01; see Fig E4 in this article's Online Repository at www.jacionline.org) that were similar to those in healthy control subjects (Fig 4, *A* and *B*). In these functional studies there is a difference between the T_FH_ activity of cells transduced using the different transduction conditions in comparison with uncorrected cells. Although an overall improvement is seen across all conditions, this is most pronounced in cells from patients with XLP transduced with the pro-T_FH_ cytokine cocktail of IL-6, IL-7, and IL-21 with significantly greater IL-21 secretion and immunoglobulin production in comparison with XLP-uncorrected cells. Cells from patients with XLP transduced with the anti-CD3/CD28 Dynabeads show increased function but did not reach statistical significance. These results demonstrate that on SAP correction, naive T cells from patients with XLP1 can be stimulated to differentiate into T_FH_ cells, which in turn are capable of providing adequate B-cell help to allow for immunoglobulin secretion *in vitro*.

### Lentivirus-mediated SAP gene transfer in CD8^+^ T cells from patients with XLP1 restores cytotoxicity

**Fig E5:**
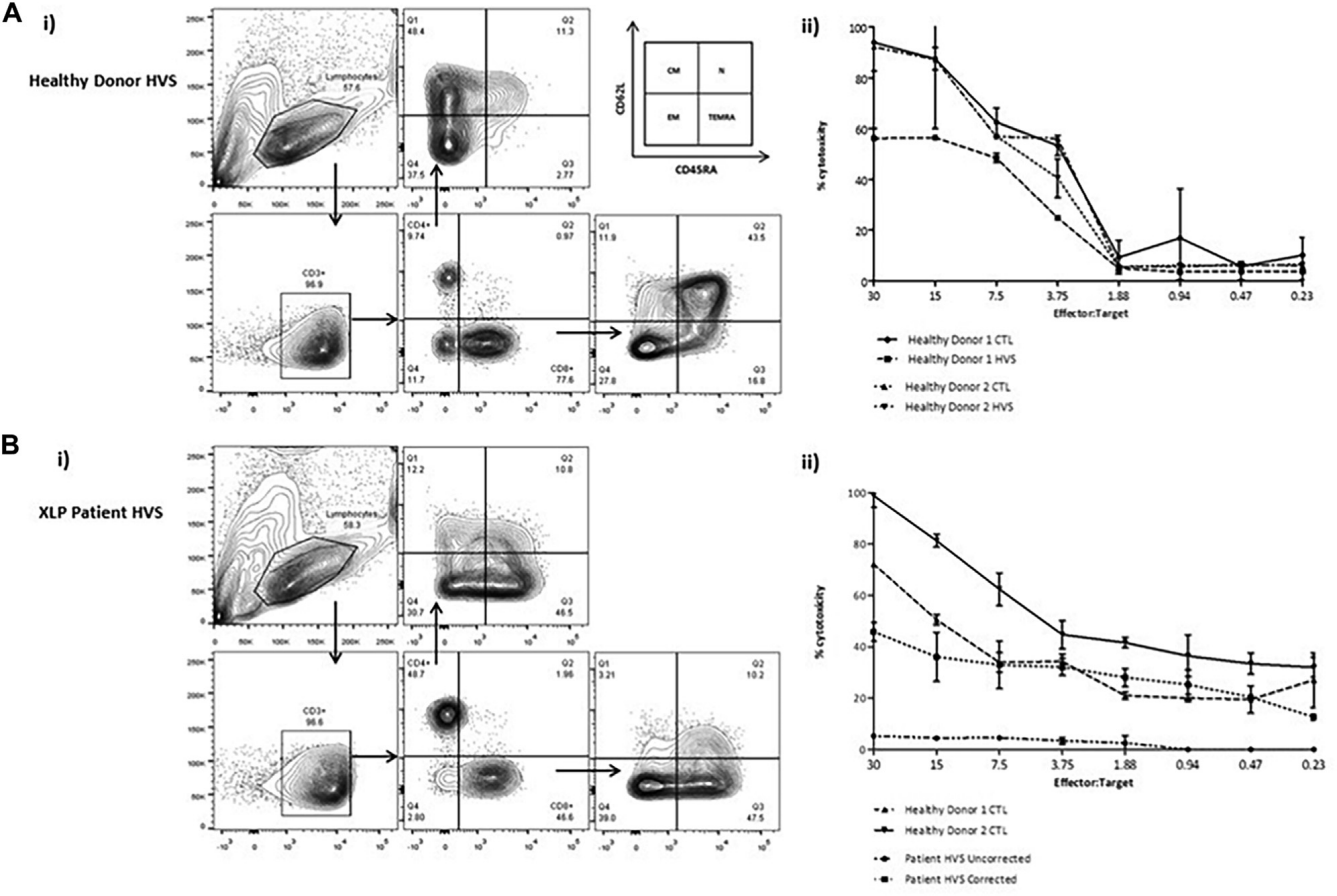
*In vitro* cytotoxicity of HVS-transformed CTLs by using an allogeneic LCL target. PBMCs from healthy donors and EBV^+^ patients with XLP were transformed with HVS. *Ai* and *Bi*, T-cell memory phenotyping was performed in both CD4 and CD8 populations to determine naive, CD45RA^+^ effector memory, effector memory, and central memory populations. Patient-transformed samples contained more terminally differentiated cells compared with healthy donors. *Aii*, ^51^Cr release assay demonstrated functional activity in HVS-transformed cells from healthy donors by using an allogeneic B-LCL target with no significant difference observed in cytotoxic activity between nontransformed and HVS-transformed cells. *Bii*, HVS-transformed cells from patients were transduced with the EFS-SAP-eGFP or EFS-eGFP mock vectors and subjected to a ^51^Cr release assay. Uncorrected patient samples showed no cytotoxic activity against allogeneic B-LCLs compared with patient-corrected CTLs, which were able to kill the LCL target within the range of 40% to 50%. No significant differences were observed between nontransformed healthy donor CTLs and patient-corrected HVS cells in cytotoxic activity when using an allogeneic B-LCL target.

SAP-deficient cytotoxic lymphocytes (CTLs) are unable to efficiently kill EBV-infected LCLs.^3^, ^29^ We investigated whether restoration of SAP expression in deficient T cells could restore cytotoxicity in this context. To examine the effect of lentivirus-mediated SAP gene transfer on cytotoxicity in human CD8^+^ EBV-specific CTLs, we again used HVS-transformed cells from both healthy donors and patients with XLP. Before gene modification, we were able to demonstrate that these transformed cells maintained cytotoxic function, with no significant difference in killing compared with EBV-CTLs derived from fresh PBMCs (from the same donor) by using a standard *in vitro* ^51^Cr release assay (see Fig E5 in this article's Online Repository at www.jacionline.org).

**Fig 5:**
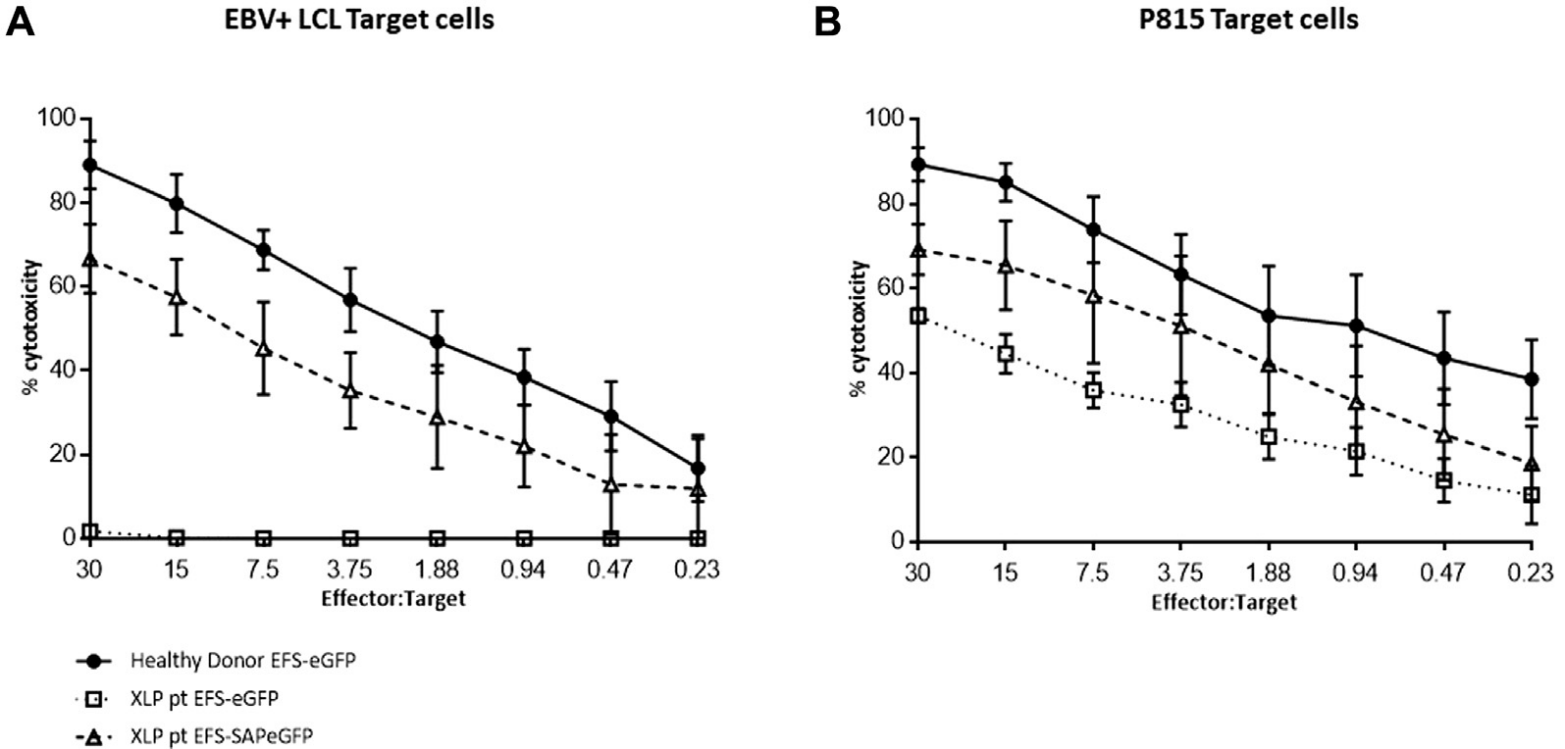
SAP gene correction of CTLs from patients with XLP1 restores cytotoxicity *in vitro*. **A,***In vitro* cytotoxic activity of CTLs generated from both healthy donors and EBV-seropositive patients with XLP1 (gene corrected and uncorrected) against allogeneic LCL target cells before intravenous infusion into NSG mice, as measured in a ^51^Cr release assay. Assays were performed in triplicates, and data shown are means ± SEMs of all values. **B,** Specificity of SAP function was determined by using non-LCL targets in a cytotoxicity assay in parallel. Murine mastocytoma P815 cells were cocultured with corrected and uncorrected effector cells from healthy donors and patients incubated with soluble anti-CD3. CTLS from all donors, including patients with XLP1, displayed cytotoxic activity, suggesting that EBV^+^ LCL-targeted killing is SAP and not CD3 mediated.

To demonstrate that this response is mediated through a SAP-dependent pathway and not through CD3, we cocultured healthy donor and noncorrected and corrected T cells from patients with XLP1 against p815 tumor cells incubated with soluble CD3 (Fig 5). All 3 lines demonstrated cytotoxicity against p815 cells, suggesting that in patients with XLP1, T-cell cytotoxicity is maintained against non-LCL targets, but expression of SAP is required for cytotoxicity against EBV-LCLs.

Patient-derived HVS cells were transduced with the EFS-SAP-eGFP vector, and EBV-CTL cytotoxicity against both autologous and allogeneic LCL targets was assessed by using a ^51^Cr release assay. Our results demonstrate recovery of killing activity in gene-corrected CTLs from patients, with transduction efficiency ranging from 24% to 48%, an average final vector copy number of 1.9 viral copies per cell (Fig 5 and see Fig E5), and no significant differences in cytotoxic function when using an autologous and allogeneic target.

### Adoptive transfer of gene-corrected EBV-CTLs from patients with XLP1 leads to regression of EBV-related lymphoma in an *in vivo*–xenografted NSG mouse model

**Fig 6:**
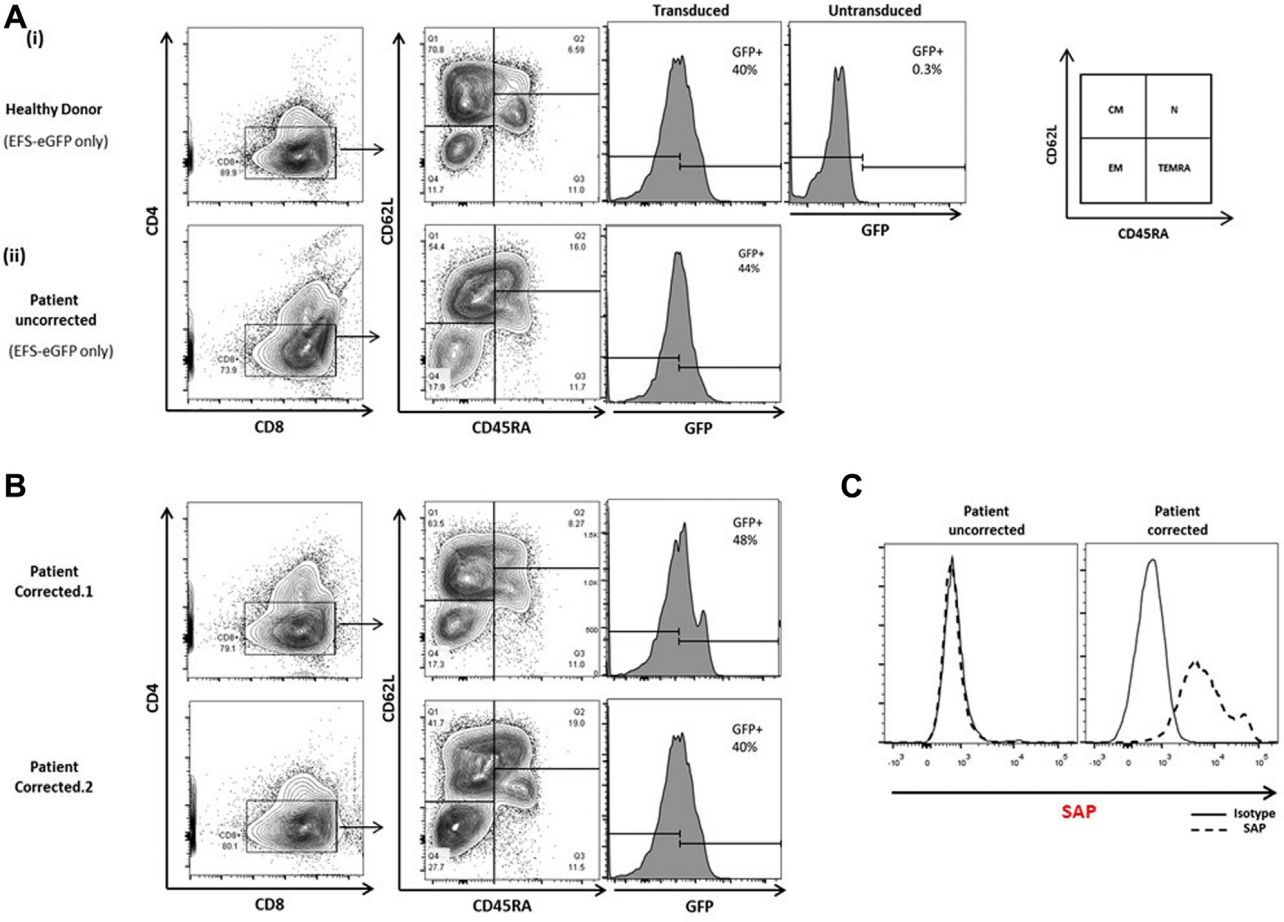
Phenotype of transduced and gene-corrected CTLs. **A,** Representative flow cytometric contour plots of CTL phenotype after *in vitro* stimulation with allogeneic LCLs in mock-transduced cells from healthy donors and patients and **B,** cells transduced with a corrective lentiviral SAP vector from patients. Transduction efficiency was assessed by using flow cytometry; eGFP expression ranged from 24% to 50%. *CM*, Central memory; *EM*, effector memory; *N*, naive; *TEMRA*, CD45RA^+^ effector memory. **C,** SAP expression from GFP^+^ selected uncorrected and gene-corrected cells from patients, as analyzed by using intracellular fluorescence-activate cell sorting staining. *Solid line*, Control IgG_2b_; *dotted line*, anti-SAP antibody.

We sought to determine whether adoptive transfer of gene-corrected T cells could mediate tumor clearance in NSG mice engrafted with EBV^+^ LCL tumors. EBV-LCLs from a healthy donor were transduced with a luciferase-expressing cassette and transplanted into NSG mice to form a palpable tumor. CTLs were generated from PBMCs from healthy donors and 2 patients with XLP1. CTLs from patients were transduced with the EFS-SAP-eGFP vector after allogeneic LCL stimulation. Seventy-two hours after transduction, cells were phenotyped, and transduction efficiency was determined by using flow cytometry (Fig 6, *A* and *B*). Phenotyping of transduced CD8^+^ CTLs demonstrated persistence of both central memory (CD45RA^−^CD62L^+^) and naive (CD45RA^+^CD62L^+^) populations across donors (range, 40% to 70% and 8% to 20%, respectively), suggesting maintenance of long-lived T-cell populations after transduction with our lentiviral vectors. Transduction efficiencies in the range of 24% to 50% were achieved at an MOI of 50, which was associated with an increase in intracellular SAP expression (Fig 6, *C*) and restoration of cytotoxic function (Fig 5).

**Fig 7:**
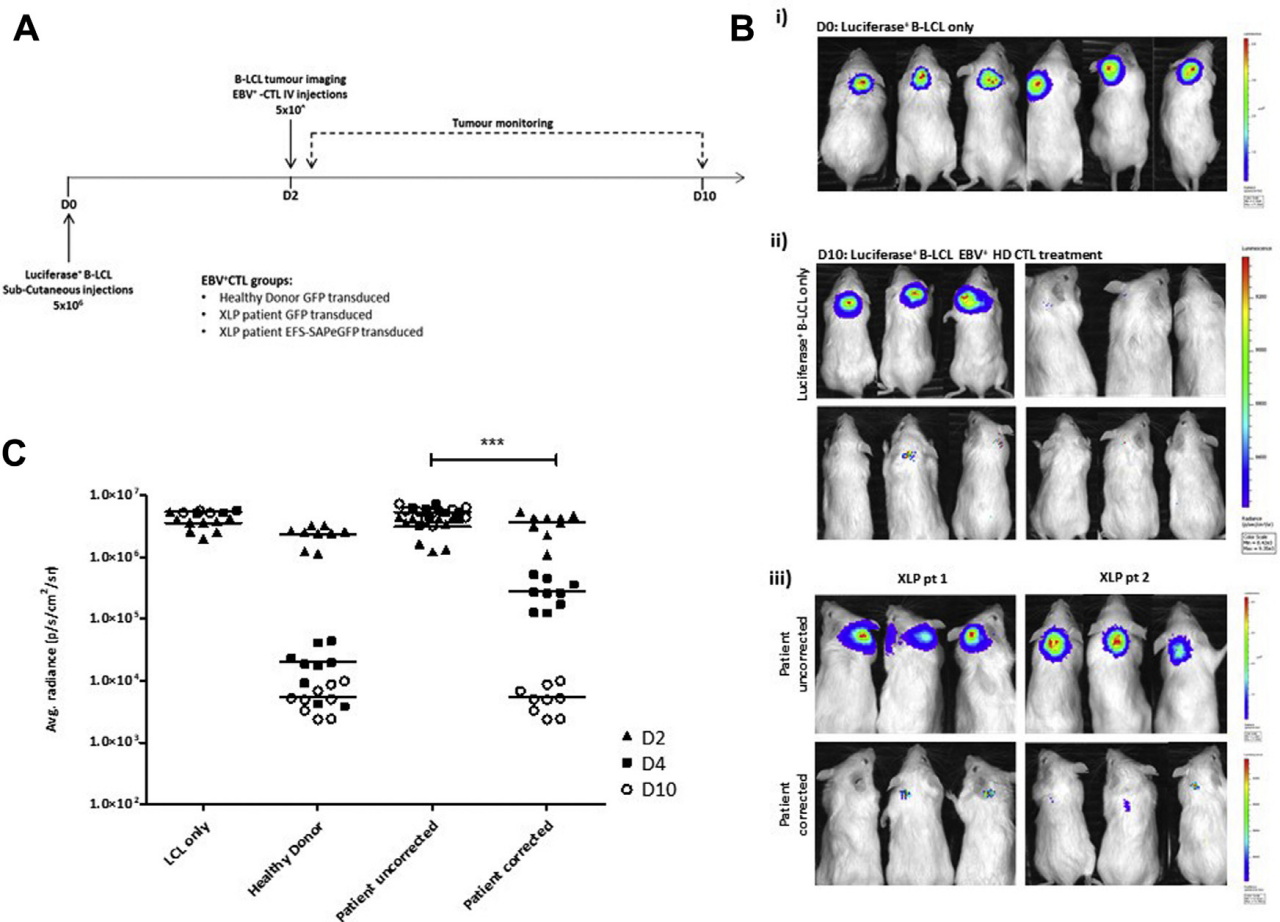
Adoptive transfer of gene-corrected CTLs from patients with XLP1 induces regression of EBV^+^ LCL-generated tumors in an NSG mouse model. **A,** EBV^+^ B-LCL xenograft tumor model experimental design. **B,***i*, Bioluminescence images of NSG mice 48 hours after subcutaneous LCL injections displaying formation of localized solid tumors. *ii*, Tumor burden after 10 days in untreated mice (*top left panel*, n = 3) and mice treated with healthy donor CTLs (3 donors, 3 animals per donor). *iii*, Tumor burden after 10 days in mice receiving uncorrected CTLs from patients with XLP1 *(top panel)* or gene-corrected CTLs from patients (*bottom panel*; 2 patient donors, 3 animals per donor) showing reduction in tumor burden in animals receiving gene-corrected cells from patients with XLP1. **C,** Dot plot representing quantification of tumor burden determined by using average photon density per second per square centimeter per steradian (p/s/cm^2^/sr) on the day of CTL infusions *(D0)*, after 48 hours *(D2)*, and after 10 days *(D10)*. ***P < .0001.

EBV-CTLs were injected intravenously 48 hours after establishment of tumors at a ratio of 1:1 LCL/CTL and bioluminescence analyzed every 48 hours subsequently (Fig 7, *A*). Regression of tumors could be visualized by day 2 after CTL infusion, with complete clearance observed in both healthy control subjects and gene-corrected patients with XLP by day 10, which is in stark contrast to animals receiving uncorrected patients' cells (Fig 7, *B* and *C*). We observed tumor persistence in untreated (n = 3) and patient-uncorrected (n = 6; EFS-eGFP–transduced) CTL-treated mice in a range of 3.0 to 6.0 × 10^6^ p/s/cm^2^/sr by day 10 after LCL infusion. In contrast, mice treated with healthy donor CTLs (n = 9, CTLs from 3 donors) demonstrated a significant reduction in tumor burden 48 hours after CTL treatment, with bioluminescence values decreasing from 3.0 × 10^6^ to 4.0 × 10^4^ p/s/cm^2^/sr and even further by day 10 to a final value of 1.0 × 10^3^ p/s/cm^2^/sr, which is equivalent to that of an animal that was not subjected to tumor engraftment. Similarly, we found a considerable reduction in tumor burden in animals infused with gene-corrected CTLs from patients (n = 6, CTLs from 2 patients) which, by day 10, was equivalent to the level of tumor clearance observed in animals treated with healthy donor CTLs and significantly better than CTL-mediated tumor clearance in animals receiving uncorrected CTLs from patients (*P* < .0001).

## Discussion

Hematopoietic stem cell gene therapy has been used successfully to treat a number of monogenic hematologic and immunologic diseases and offers a curative treatment option for patients lacking a suitable donor for HSCT. XLP1 is a monogenic primary immune deficiency with a range of severe manifestations, and even in cases of early diagnosis, provision of prophylactic therapies, and close monitoring, the condition can be fatal because of the development of HLH or malignancy.^10^, ^30^ Outcomes after HSCT from mismatched donors is significantly worse in this population than from HLA-identical donors,^10^ and as such, development of novel gene therapy strategies can offer patients lacking suitable donors an alternative management option. We have previously described correction of a Sap-deficient murine model using gene-corrected HSCs with *SAP* transgene expression controlled by the ubiquitously active EFS promoter,^12^ but given the role of SAP as an intracellular signaling molecule and the lack of normal SAP expression in HSCs, we explored use of SAP gene transfer in T cells, in which it is normally expressed as an alternative option.

Here we provide the first evidence that adoptive transfer of gene-corrected T cells can correct the humoral and cytotoxic defects associated with XLP1; this could represent a clinically applicable therapy for patients with this condition. Immune abnormalities in patients with XLP1 arise predominantly from defects in the T-cell compartment. Therefore correction of CD3^+^ lymphocytes can afford protection from HLH caused by dysregulated T-cell activation and impaired cytotoxicity alongside reduced risk of malignancy through improved tumor surveillance and improved humoral immunity through correction of T_FH_ cell function. Although the NK cell population will remain uncorrected, there is evidence of redundancy in the role these cells play in the pathophysiology of HLH, and therefore correction of the CD8 compartment alone might be sufficient.^31^

In an established Sap-deficient murine model, we have shown *in vivo* that transfer of SAP-corrected T lymphocytes leads to recovery of functional humoral immunity after T cell–dependent antigen challenge through formation of germinal centers and specific antibody responses, both of which are absent in Sap-deficient animals. This is achievable at levels of engraftment close to 40%, which is clinically feasible with current lentiviral transduction platforms in human primary cells. Moreover, we were able to demonstrate functional correction of T_FH_ cells from patients *in vitro* with significantly improved IL-21 and immunoglobulin secretion profiles. The functional profile of gene-corrected T_FH_ cells from patients with XLP was enhanced when cells were cultured with IL-6, IL-7, and IL-21, cytokines chosen based on their role in the differentiation and maintenance of the T_FH_ cell population.^27^ Together, these data suggest that T-cell gene therapy could be of considerable clinical benefit to patients with XLP1 with dysgammaglobulinemia.

Our data also confirm that lentivirus-mediated SAP gene transfer can correct cytotoxic defects in CD8^+^ CTLs from patients in the context of EBV, which is essential in preventing and treating HLH and lymphoproliferative complications. This has been demonstrated through both *in vitro* cytotoxicity assays and an *in vivo* EBV-LCL lymphoma tumor model in NSG mice. We show convincingly that gene-corrected CTLs from patients are able to induce tumor regression to the same level as CTLs from healthy donors. Therefore we can assume that gene-modified CD8^+^ T cells from patients would be functional in the context of EBV viral challenge and potentially HLH in patients with XLP1.

The anticipated level of engraftment of gene-corrected T cells required to permit functional immune reconstitution in patients is an important question, but there is limited clinical experience of patients after HSCT with low-level mixed chimerism. We have shown here that T lymphocytes from patients can be transduced efficiently, leading to restoration of SAP expression, and although levels of expression are less than those seen in healthy donor cells, a modest increase in protein levels correlates with correction of T_FH_ cell function and cytotoxicity. Although it is challenging to define the exact level of engraftment required, we can extrapolate from the data presented here that as little as 15% correction can restore immune function. Taken in the clinical context, these results are encouraging, suggesting that low levels of engraftment might be sufficient for phenotype rescue.

The long-term persistence of autologous gene-modified T cells and thus the longevity of clinical benefit also remains to be determined. Early clinical trials for adenosine deaminase–deficient severe combined immune deficiency used peripheral T cells transduced with a gammaretroviral vector, and gene-marked cells were detectable more than 10 years after infusion.^32^ Similar findings have also been reported in the context of suicide gene therapy clinical trials.^31^ It has now been established that specific T-cell subsets are capable of sustaining the T-cell compartment, namely stem cell memory T and central memory T cells.^33^ Here we have shown maintenance of a central memory population after transduction with our lentiviral construct, supporting the idea that SAP-corrected cells from patients can persist for several years. Whether T-cell gene therapy offers a definitive treatment for patients with XLP1 lacking a donor for transplantation will only become evident through a clinical trial. Although a T-cell strategy should provide long-term benefit, it also does not prevent patients from receiving a subsequent stem cell procedure and in this context can also be used to treat patients with XLP1 before HSCT if a suitable donor is unavailable within the necessary time period.

In conclusion, the data presented here strongly support clinical translation of a lentivirus-mediated T-cell gene therapy approach to treat patients with XLP1 who lack a suitable donor for HSCT. Given that we have shown amelioration of both humoral and cellular immune defects, patients with a range of clinical phenotypes can benefit from this therapeutic strategy.

**Key message**

• This study demonstrates that adoptive transfer of gene-corrected T cells corrects humoral and cytotoxic abnormalities seen in patients with XLP1, establishing potential therapeutic benefit of this approach for patients with XLP1.

## Methods

### Vector copy number and Y chromosome engraftment analysis

Genomic DNA was extracted from either human cultured CD4/CD8 cells or murine splenic CD3^+^ cells by using the Qiagen DNeasy blood and tissue extraction kit, according to the manufacturer's protocol. Multiplex quantitative PCR with gBLOCK (standards) synthesized and purchased from Integrated DNA Technologies was carried out to determine vector copy number and murine donor T-cell engraftment.

### Generation of HVS-transformed human primary T cells and cytotoxicity

PMBCs from healthy donors or patients with XLP1 were stimulated *in vitro* with 20 μg/mL PHA and then cultured in X-VIVO media with 5% human serum for a period of 72 hours. After 72 hours, IL-2 (20 ng/mL) was added to supplement media, and cells were incubated for a further 48 hours. HVS viral supernatant generated by using owl monkey kidney cells, as previously published, was added to cultured cells seeded at a density of 2.0 × 10^6^ cells/well of a 48-well plate. The phenotype of transformed cells was assessed at 2-week intervals to determine cell longevity and viability.

### Flow cytometry, immunophenotype, and SAP staining

Single-cell suspensions were prepared from human PBMCs or murine splenic tissue. Samples were incubated for 60 minutes at room temperature with various antibodies: BV421 rat anti-human CD185 (CXCR5) BV510, mouse anti-human CD279 (PD-1) or Alexa Fluor 647 rat anti-mouse GL7 PE rat anti-mouse CD19, PE rat anti-mouse CXCR5, or BV421 hamster anti-mouse CD279 (PD-1) and analyzed by using flow cytometry for GFP and cell-surface marker expression. For SAP intracellular staining, after fixation and permeabilization (IntraPrep; Beckman Coulter, Fullerton Calif), cells were incubated either with a mouse anti-human SAP (SH2D1A) antibody (clone 1C9; Abnova, Jhongli, Taiwan) or the corresponding isotype control Rat IgG_1_, κ (clone R3-34 RUO; BD PharMingen).

### Immunization and antibody response

For immunization, animals received intraperitoneal injections of NP(65)-CGG (150 μg/mL per mouse; Biosearch Technologies, Novato, Calif) mixed with alum (Serva, Heidelberg, Germany) in a 1:2 ratio. Serum was collected through a tail vein bleed at various time points after immunization, and NP-specific immunoglobulins were detected and quantitated by using ELISAs with NP(23)-BSA (Biosearch Technologies) as a capture antigen. The mouse immunoglobulin standard panel was obtained from SouthernBiotech, Birmingham, Ala.

To analyze germinal center formation, immunized animals were killed 10 days after NP-CGG injection, and germinal center B cells were detected by using flow cytometry in single-cell suspensions of splenocytes after erythrocyte lysis with anti-CD19 and anti–GL-7 antibodies (BD Biosciences). Spleens were then fixed in formalin, sectioned, and stained with hematoxylin and eosin to allow visualization of tissue structure. Germinal center B cells were stained by means of immunohistochemistry with unconjugated peanut agglutinin (Vector Laboratories, Burlingame, Calif). Images were captured with Olympus BX51 with Olympus Plan Apo objectives (Olympus, Center Valley, Pa), and the Nikon DigitalSight DS-L1 digital microscope camera (Nikon, Tokyo) with automatic exposure and on-camera white balancing. Magnifications are as stated in the figures and figure legends.
